# Apoptotic markers in cultured fibroblasts correlate with brain metabolites and regional brain volume in antipsychotic-naive first-episode schizophrenia and healthy controls

**DOI:** 10.1038/tp.2015.122

**Published:** 2015-08-25

**Authors:** A Batalla, N Bargalló, P Gassó, O Molina, D Pareto, S Mas, J M Roca, M Bernardo, A Lafuente, E Parellada

**Affiliations:** 1 Department of Psychiatry and Psychology, Clinical Institute of Neuroscience, Hospital Clínic de Barcelona, Barcelona, Spain; 2 Department of Psychiatry and Clinical Psychobiology, University of Barcelona, Barcelona, Spain; 3 Radboud University Medical Centre, Department of Psychiatry, Nijmegen, The Netherlands; 4 Radboud University, Nijmegen Institute for Scientist-Practitioners in Addiction, Nijmegen, The Netherlands; 5 Medical Image Core facility Institut d'Investigacions Biomèdiques August Pi i Sunyer, Centre de diagnòstic per la Imatge Clínic, Hospital Clinic of Barcelona, Barcelona, Spain; 6 Institut d'Investigacions Biomèdiques August Pi i Sunyer, Barcelona, Spain; 7 Centro de Investigación Biomédica en Red de Salud Mental, Barcelona, Spain; 8 Department of Pathological Anatomy, Pharmacology and Microbiology, University of Barcelona, Barcelona, Spain; 9 Department of Psychiatry, Hospital Universitari Mútua de Terrassa, Barcelona, Spain; 10 Magnetic Resonance Unit, Vall Hebron University Hospital IDI, Barcelona, Spain; 11 Barcelona Clinic Schizophrenia Unit, Neuroscience Institute, Hospital Clinic of Barcelona, Barcelona, Spain

## Abstract

Cultured fibroblasts from first-episode schizophrenia patients (FES) have shown increased susceptibility to apoptosis, which may be related to glutamate dysfunction and progressive neuroanatomical changes. Here we determine whether apoptotic markers obtained from cultured fibroblasts in FES and controls correlate with changes in brain glutamate and *N*-acetylaspartate (NAA) and regional brain volumes. Eleven antipsychotic-naive FES and seven age- and gender-matched controls underwent 3-Tesla magnetic resonance imaging scanning. Glutamate plus glutamine (Glx) and NAA levels were measured in the anterior cingulate (AC) and the left thalamus (LT). Hallmarks of apoptotic susceptibility (caspase-3-baseline activity, phosphatidylserine externalization and chromatin condensation) were measured in fibroblast cultures obtained from skin biopsies after inducing apoptosis with staurosporine (STS) at doses of 0.25 and 0.5 μM. Apoptotic biomarkers were correlated to brain metabolites and regional brain volume. FES and controls showed a negative correlation in the AC between Glx levels and percentages of cells with condensed chromatin (CC) after both apoptosis inductions (STS 0.5 μM: *r*=−0.90; *P*=0.001; STS 0.25 μM: *r*=−0.73; *P*=0.003), and between NAA and cells with CC (STS 0.5 μM induction *r*=−0.76; *P*=0.002; STS 0.25 μM *r*=−0.62; *P*=0.01). In addition, we found a negative correlation between percentages of cells with CC and regional brain volume in the right supratemporal cortex and post-central region (STS 0.25 and 0.5 μM; *P*<0.05 family-wise error corrected (FWEc)). We reveal for the first time that peripheral markers of apoptotic susceptibility may correlate with brain metabolites, Glx and NAA, and regional brain volume in FES and controls, which is consistent with the neuroprogressive theories around the onset of the schizophrenia illness.

## Introduction

Dopaminergic abnormalities are the core feature of psychosis; however, there are several aspects of schizophrenia that do not appear to be entirely explained by dopamine dysfunction alone.^1^ Clinical research has suggested that abnormalities in dopaminergic neurotransmission may be the final common pathway to psychosis,^2^ and this may be driven by abnormalities in glutamatergic transmission.^3, 4, 5^ The glutamatergic hypothesis of schizophrenia was proposed in the early 1990s,^6^ based on pharmacological studies with N-methyl-D-aspartate receptor blockers, such as phencycline and ketamine, which produce psychotomimetic effects in healthy volunteers and exacerbate symptoms and cognitive defects in schizophrenia patients.^7, 8^ Normal glutamate (Glu) and N-methyl-D-aspartate receptor function has a critical role in synaptogenesis during early development and synaptic elimination during adolescence.^9^ The administration of N-methyl-D-aspartate receptor antagonists to experimental animals during early postnatal development results in cortical apoptosis, and behavioral, structural and neurochemical abnormalities associated with schizophrenia.^10, 11, 12^ Deficits in glutamatergic neurotransmission, including NMDA receptor hypofunction and Glu-induced excitotoxicity, are capable of activating apoptosis in neurons, which may lead to neuronal damage and the onset of psychosis in early adulthood.^13, 14^

First-episode schizophrenia patients may have a genetically increased susceptibility to apoptosis or be more vulnerable to pro-apoptotic stimuli, such as Glu excitotoxicity, oxidative stress, reduced neurotrophic support and inter alia.^13, 15^ This increased apoptosis might explain certain features of schizophrenia, such as the progressive gray matter (GM) loss identified in first-episode schizophrenia (FES), or the functional brain deficits observed in the course of the disease.^16, 17^ Post-mortem studies do not show clear evidence of decreased cell viability, but local synaptic apoptosis may reduce neuropil without inducing neuronal death.^18^ Reduced dendritic spines, presynaptic marker proteins and expression of synaptic genes^13, 19^ are consistent with the hypothesis of loss of neuropil or altered apoptosis.^13, 15, 20^

*In vivo* measures of glutamate metabolism and neural integrity may help to test glutamatergic models of schizophrenia and its relation to apoptotic markers.^21^ Few studies have examined the levels of Glu, its metabolite glutamine (Gln) or its combination (Glx), as well as *N*-acetylaspartate (NAA), a marker of neuronal integrity, using proton magnetic resonance spectroscopy (1H-MRS) in first-episode psychosis before treatment.^7, 22, 23^ Two 1H-MRS studies in unmedicated first-episode patients found increased Gln/Glu ratio^7^ and Gln levels^23^ in the anterior cingulate (AC), and another in the medial prefrontal cortex.^22^ Increased Gln has also been reported in the thalamus in one study,^23^ as well as decreased NAA in the AC.^7^ Studies involving clinical high-risk populations have also described high levels of Gln in cortical regions,^24, 25^ although lower^26^ and negative results have also been reported.^27, 28^ Importantly, the loss of thalamic Gln in patients with schizophrenia^29^ and Glu in subjects at risk of developing schizophrenia^24^ has been correlated with GM loss in several brain regions.

On the basis of evidence that glutamatergic dysfunction may lead to cortical apoptosis, and that the loss of Glu, Gln and NAA may be related to loss of synaptic neuropil and structural abnormalities in different stages of psychotic illness, we hypothesized that changes in Glx and NAA measured with 1H-MRS would correlate with apoptotic markers obtained from cultured fibroblasts in antipsychotic-naive first-episode psychotic patients compared with healthy controls. Several markers enable to detect morphological, biochemical and molecular alterations in cells undergoing apoptosis. One of the earliest markers of this programmed cell death involves the externalization of phosphatidylserine residues from the inner to the outer leaflet of the cell membrane.^30^ Later stages involve the activation of caspases, caspase-3 being the ultimate and main executor of apoptosis, as well as chromatin condensation and DNA fragmentation.^31, 32^ We further hypothesized that apoptotic markers would correlate with regional brain volume alterations measured with voxel-based morphometry (VBM), particularly in those areas that may be expected to be involved in schizophrenia, such as the frontal and temporal cortex.

## Materials and methods

### Subjects

Eleven patients (six men and five women) suffering from a FES and aged between 19 and 29 years were recruited during acute hospitalization for a first psychotic episode in the inpatient unit of a general academic hospital (Hospital Clinic, Barcelona) over a period of 2 years. All patients were antipsychotic-naive at the time of sample collection. Psychopathology and functionality of patients were assessed with the Positive and Negative Syndrome Scale (PANSS), the Clinical Global Impression (CGI) and the Global Assessment of Functioning (GAF) scales. The diagnosis of schizophrenia was confirmed according to Diagnostic and Statistical Manual of Mental Disorders, 4th Edition, Text Revision after a 2-year follow-up clinical evaluation. Seven healthy controls (four men and three women), college students from the University of Barcelona (age range 21–26 years), were also recruited at the same time. All subjects included in the study were Caucasians, except for one patient who reported African ancestry. Control subjects were assessed using a semistructured psychiatric interview (SCID-I), excluding any Axis I psychiatric disorders. Subjects suffering from mental retardation or from any neurological illness were excluded. No subjects suffered from any relevant disease. Chronic drug treatment was considered a criterion for exclusion. People meeting Diagnostic and Statistical Manual of Mental Disorders, 4th Edition criteria for substance abuse or dependence for any substance save nicotine or cannabis and alcohol used sporadically were also excluded. Demographic and clinical data are shown in Table 1. After receiving a full explanation of the study, written informed consent was obtained from each control subject and, in the case of patients, from their parents. The study was approved by the Ethics Committee of Hospital Clínic. Two subjects (one patient and one control) could not finish the exam and the spectra were not acquired.

### Image acquisition and analysis

All images were acquired at a 3 T Siemens Magneto TIM Trio (Siemens Diagnostics Healthcare, Erlangen, Germany) at the Image Platform of IDIBAPS, Centre de diagnostic per la Imatge from Hospital Clínic, Barcelona. We used a 32-channel phased-array head coil with foam padding and headphones to restrict head motion and suppress scanner noise.

### 1H-MRS acquisition and quantification

**Figure 1:**
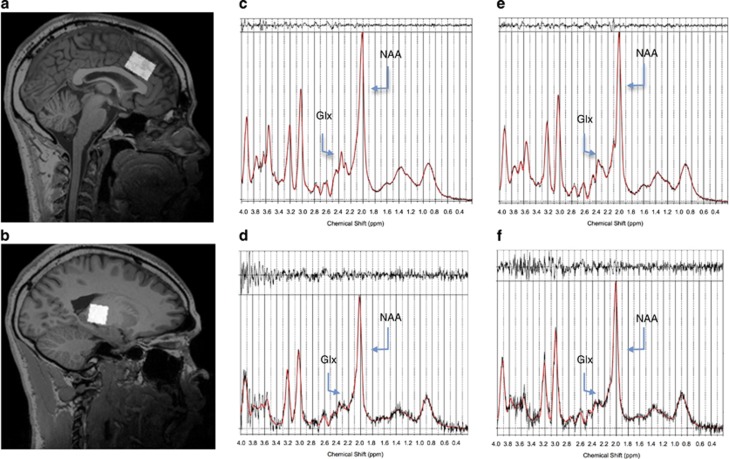
Spectroscopic voxel placement in the anterior cingulate (AC) cortex (**a**) and in the left thalamus (LT; **b**). Representative spectra of one control in the AC (**c**) and in the LT (**d**) and of one patient in the AC (**e**) and in the LT (**f**). Glx, glutamate+glutamine; NAA, *N*-acetylaspartate.

Single voxel spectra were acquired with the use of a double-spin echo point-resolved spectroscopy sequence, with repetition time=1500 ms and echo time=35 ms, data points 2048, with automatic shimming and water suppression. A volume of interest (VOI) of 12 cm^3^ was placed in the AC (30 × 20 × 20 mm^3^), and a second one of 8 cm^3^ in the left thalamus (LT; 20 × 20 × 20 mm^3^). Figure 1 demonstrates representative voxel placement. This procedure was applied in the same manner in all subjects, and care was taken to ensure standard placement. For the quantification, we used the user-independent frequency-domain fitting program (LC Model)^33, 34^ version 6.1-4A, using a basis set of model metabolite spectra. Eddy-current correction was conducted. The unsuppressed water signal was used as an internal reference, assuming a water concentration of 35 880 mmol l^−1^ (ref. 35) to estimate the metabolite concentrations. No corrections for relaxation were performed. Therefore, the concentrations are expressed as internal units that differ from mmol kg^−1^ by factors because of relaxation, as well as water content in the VOI. Some metabolites are difficult to quantify independently from others; therefore, the sum of concentrations was used. The metabolites of the basis set for LC Model were L-Alanine (Ala), Aspartate (Asp), Cr, *gamma*-Aminobutyric acid, Glu, Gln, inositol (mI), L-Lactate (Lac), NAA, *N*-acetyl aspartateglutamate (NAAG), Scyllo-Inositol (Scyllo), Taurine (Tau), Glycerophosphocholine (GPCh), Phosphocholine (PCh), Glycine (Gly), the combined metabolites (GPCh+PCh, NAA+NAAG and Glu+Gln) and the different groups of lipids and macromolecules (Lip13a, Lip13b, Lip09, MM09, Lip20, MM20, MM12, MM14 and MM17) and their combinations (Lip13a+Lip13b, MM14+Lip13a+Lip13b+MM12, MM09+Lip09 and MM20+Lip20). Only the metabolites NAA, NAAG, Glu, Gln, Cr, GPCh, PCh and mI, some of them combined (GPCh+PCh referred to as total Cho, NAA+NAAG referred to as total NAA and Glu+Gln referred to as Glx) were considered as they are the ones that have been studied the most in the previous schizophrenia literature. A typical LCM spectrum and fitting for both patients and controls are shown in Figure 1. We only consider the absolute metabolite values with a Cramer–Rao lower bound below 20%, indicating that these metabolites could be reliably estimated,^34^ and a signal-to-noise ratio greater than 10. We decided to use the sum Glu and Gln because these metabolites separately usually do not survive the Cramer–Rao lower bound level.

An additional structural image (3d T1-weighted MPRAGE sequence with isometric voxel of 1x1x1 mm^3^) was recorded in the same scanning session (the sequence is described below). Statistical parametric mapping (SPM) segmentation was performed and cerebrospinal fluid was included in the VOI (SPM5 software, running in Matlab 6.5, MathWorks, Natick, MA, USA). Metabolite concentrations were adjusted for the amount of cerebrospinal fluid in each voxel. The metabolite concentrations were adjusted for the amount of cerebrospinal fluid contained within the VOI using the formula: 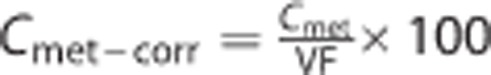 where VF corresponds to the volume fraction (percentage of nervous tissue − GM and white matter − contained in the VOI).^36^ The 1H-MRS parameters used for the present study provided robust signals for both the healthy control and FES groups. Specifically, healthy controls had an AC and LT signal-to-noise ratio of 68.50 (s.d.=9.75) and 15.00 (s.d.=2.68), respectively, and a full-width at half-maximum of 0.049 p.p.m. (s.d.=0.013) and 0.079 p.p.m. (s.d.=0.022), respectively. FES patients had an AC and LT signal-to-noise ratio of 66.60 (s.d.=14.58) and 35.90 (s.d.=55.66), respectively, and a full-width at half-maximum of 0.042 p.p.m. (s.d.=0.011) and 0.079 p.p.m. (s.d.=0.016), respectively. None of these measures was different between the two groups (*P*>0.05), suggesting that the quality of the data was comparable across the two groups.

### Structural volumetric acquisition and VBM analysis

The magnetic resonance imaging protocol included a set of magnetization-prepared rapid gradient echo T1-weighted images (repetition time: 2300 ms; echo time: 3 ms; flip angle: 15° field of view: 245 mm; and voxel size: 1 × 1 × 1 mm^3^) and axial T2 images (repetition time: 3000 ms; echo time: 87 ms; flip angle: 120º field of view: 233 × 256; and slice thickening: 3 mm). A neuroradiologist (NB) confirmed that all magnetic resonance imaging scans were free of gross structural abnormalities.

The structural MPRAGE images were analyzed with the Statistical Parametric Mapping software (SPM8; The Wellcome Department of Imaging Neuroscience, London, UK) following the VBM toolbox (http://dbm.neuro.uni-jena.de/vbm/). Briefly, images were segmented and normalized to the SPM-T1 template using a high-dimensional DARTEL transformation. In addition, the Jacobian determinants derived from the spatial normalization were used to modulate image voxel values to restore volumetric information (affine and nonlinear).^37^ Finally, images were smoothed with an 8-mm full-width at half-maximum isotropic Gaussian kernel.

Differences between patients and controls were assessed for both gray and white matter portions, as a two-sample *t*-test. Data were generated at *P*<0.001 uncorrected, and only those surviving a cutoff of *P*<0.05, family-wise error (FWE) correction at the cluster level for multiple comparisons, were considered statistically significant. Finally, correlations with apoptotic markers were also analyzed for both gray and white matter VBM, using the same statistical threshold.

In order to assess whether there was a correlation between the metabolites (corrected by cerebrospinal fluid factor) and GM concentration, a multivariate analysis was performed. Total intracranial volume was included as a covariate. Significance level was set at *P*<0.05 (FWE-corrected, FWEc).

### Fibroblast primary cell culture: markers of apoptotic susceptibility

Hallmarks of apoptotic susceptibility (caspase-3 activity, phosphatidylserine externalization and chromatin condensation (CC)) were measured in fibroblast cultures obtained from skin biopsies of all subjects after inducing apoptosis with staurosporine (STS) at doses of 0.25 and 0.5 μM (Sigma-Aldrich, St Louis, MO, USA). Five markers of apoptotic susceptibility were considered in the analysis: caspase-3 activity (baseline and 6 h after treatment with STS 0.50 μM), externalization of phosphatidylserine (measuring annexin-V 6 h after treatment with STS 0.50 μM) and chromatin condensation (6 h after STS induction at doses of 0.25 and 0.5 μM). These biomarkers were chosen on the basis of previous results of increased apoptotic susceptibility in cultured fibroblasts from antipsychotic-naive FES patients compared with healthy controls.^15^ Compared with controls, cultured fibroblasts from patients showed higher caspase-3 activity and lower BCL2 expression. When exposed to STS, fibroblasts from patients also showed higher cleaved caspase-3 activity and protein levels; a higher percentage of cells with translocated phosphatidylserine and condensed chromatin (CC); and higher p53 expression.^15^ The detailed protocol for the establishment of the skin fibroblast cultures can be found elsewhere.^15^

### Statistical analysis

All statistical analyses were performed using Statistical Package for the Social Sciences (SPSS, v.19; SPSS, Chicago, IL, USA). Descriptive results are presented as the mean (s.d.) for continuous variables and frequencies (absolute and relative) for categorical variables. Group differences in demographic were explored using *X*^2^-test, Student's *t*-test or Mann–Whitney *U*-test, as appropriate. Metabolite measures between groups were compared using a multivariate regression analysis. The percentages of GM in the AC and LT were included as covariates. Statistical significance was set at 0.05.

Partial correlations controlling for percentages of GM and for the time of experiment when the apoptotic hallmarks were measured were conducted to examine the relationship between apoptotic markers and metabolite concentrations for each region. The statistical threshold was Bonferroni-adjusted (*P*=0.05/5=0.01) to control for multiple comparisons. Statistical power was calculated according to the recruited sample size. When all participants were included in the test (*n*=16), assuming a 5% level of significance, we were able to detect correlation coefficients of *r*⩾0.65 with ⩾80% of statistical power; when sample was stratified according to diagnosis, we were able to detect *r*⩾0.79 (cases *n*=10, 5% level of significance and ⩾80% statistical power) or *r*⩾0.92 (controls *n*=6, 5% level significance and ⩾80% statistical power).

## Results

### Cerebral metabolites

There were no significant differences between groups in Glx and NAA levels either in the AC (controls (mean, s.d.): 9.2, 1.1; patients: 7.8, 1.1; F=4.73, *P*=0.149; and controls: 7.3, 1.1; patients: 6.5, 0.8; F=1.72, *P*=0.339, respectively), or in the LT (controls (mean, s.d.): 5.4, 1.7; patients: 4.8, 1.3; F=2.08, *P*=0.602; and controls: 7.9, 1.0; patients: 7.5, 0.4; F=1.21, *P*=0.186, respectively). We found no differences in the other metabolite levels (Inositol, Cr and PCh) between groups in the AC or LT.

### Relationship between apoptotic markers and metabolite levels

#### Anterior cingulate

**Figure 2:**
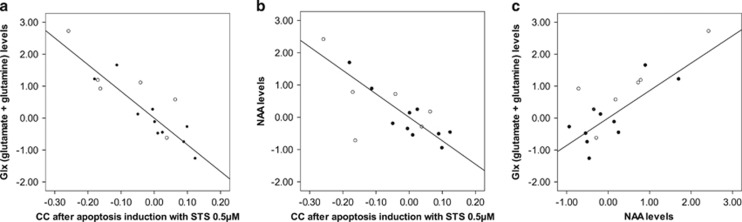
Partial correlation plots showing the association between the residualized values of apoptotic markers and metabolite concentrations after controlling for percentages of gray matter and for the time of experiement when the apoptotic hallmarks were measured. (**a**) Inverse correlation between anterior cingulate Glx (glutamate+glutamine) levels and chromatin condensation (CC) after apoptosis induction with staurosporine (STS) 0.5 μM in first-episode schizophrenia and controls (*r*=−0.90; *P*=0.001). (**b**) Inverse correlation between anterior cingulate *N*-acetylaspartate (NAA) levels and CC after apoptosis induction with STS 0.5 μM in first-episode schizophrenia and controls (*r*=−0.62; *P*=0.01). (**c**) Positive correlation between anterior cingulate glutamate+glutamine (Glx) and NAA levels in first-episode schizophrenia and controls (*r*=0.80; *P*=0.001). ●, First-episode schizophrenia; ○, controls.

There was a negative correlation between Glx levels and the percentages of cells with CC after both apoptosis inductions: STS 0.5 μM: *r*=−0.90; *P*=0.001; STS 0.25 μM: *r*=−0.73; *P*=0.003 (Figure 2). When the analysis was performed by group, we found a negative correlation in the FES group after induction with STS 0.5 μM (*r*=−0.90; *P*=0.002) that did not reach corrected significance in controls (STS 0.5 μM: *r*=−0.94; *P*=0.045). NAA levels were also negatively correlated with percentages of cells with CC after induction with STS 0.25 μM (*r*=−0.62; *P*=0.01) and 0.5 μM (*r*=−0.76; *P*=0.002; Figure 2). In the analysis by group, negative correlations were significant in FES after both apoptosis inductions (STS 0.5 μM: *r*=−0.88; *P*=0.004; and STS 0.25 μM: *r*=−0.89; *P*=0.003) but not in controls (STS 0.5 μM: *r*=−0.73; *P*=0.266; and STS 0.25 μM: *r*=−0.24; *P*=0.747). No further correlations were found in the AC between Glx or NAA levels and other apoptotic biomarkers.

Glx and NAA levels were positively correlated in the AC (*r*=0.80; *P*=0.001) of all participants (Figure 2). When data were analyzed by group, the correlation was significant in the FES group (*r*=0.77; *P*=0.026) but not in controls (*r*=0.84; *P*=0.159).

#### Left thalamus

We found two negative correlations between Glx and levels of caspase-3 activity (both baseline and 6 h after treatment with STS 0.50 μM) that did not overcome the correction for multiple comparisons (baseline *r*=−0.59; *P*=0.034; STS 0.5 μM *r*=−0.56; *P*=0.047). No further correlations were found in the LT between Glx or NAA levels and other apoptotic biomarkers.

### Relationship between apoptotic markers and regional brain volume

**Figure 3:**
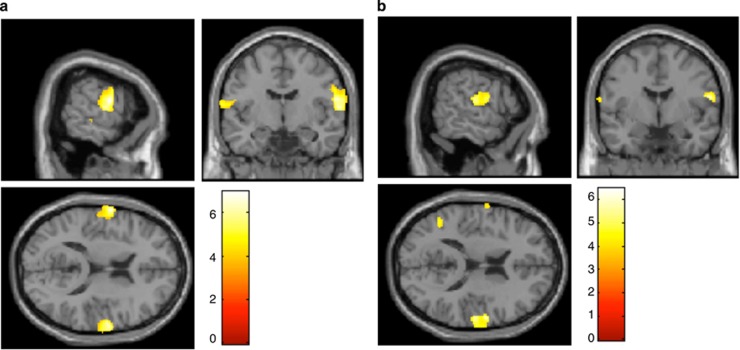
Correlation of regional brain volume with chromatin condensation (CC) after apoptosis induction with staurosporine (STS) in first-episode schizophrenia (FES) and controls. (**a**) Significant negative correlation in the right superior temporal and in the right postcentral after apoptosis induction with STS 0.25 μM (*P*<0.001, family-wise error (FWE)-corrected at a cluster level). (**b**) Significant negative correlation in the right post-central region after apoptosis induction with STS 0.5 μM (*P*<0.001, FWE-corrected at a cluster level). Images are superimposed on selected slices of a normalized brain and are oriented following the neurological convention (right=right). Voxels with *P*<0.001 (uncorrected) are displayed. Color bar represents *t*-value.

We did not find significant gray and white matter differences between patients and controls. A negative correlation between regional brain volume in the right superior temporal cortex and in the postcentral gyrus and percentages of cells with CC after both apoptosis inductions (STS 0.25 and 0.5 μM) was found (*P*<0.05 FWEc; Figure 3). The analysis by group could not be performed, given that the sample size was lower than the degrees of freedom needed. No other correlations between gray and white matter volume and apoptotic biomarkers were found.

### Relationship between metabolite levels and regional brain volume

None of the metabolites considered showed a significant positive or negative correlation with gray matter.

## Discussion

To the best of our knowledge, this is the first neuroimaging study to examine the relationship between brain metabolite levels and regional brain volume and peripheral apoptotic biomarkers in first-episode antipsychotic-naive schizophrenia patients and controls. Our results showed a negative correlation in the AC between Glx and NAA levels and percentages of cells with CC in cultured fibroblasts obtained from skin biopsies. We further show that regional brain volume in the right superior temporal cortex and in the postcentral region was negatively correlated to the same apoptotic biomarker. Taken together, these findings are consistent with the neuroprogressive theories around the early stages of the illness.

One of the defining neuropathological features of schizophrenia is GM loss in frontal and temporal regions, which is accelerated during periadolescence.^38^ There has been substantial controversy about what causes the progressive changes. Possible explanations include the effects of medication,^39^ excitotoxicity induced by N-methyl-D-aspartate Glu receptor dysfunction^40^ and reduced neuropil,^20^ which may be influenced by local synaptic apoptosis.^15^ In our sample of untreated FES patients, we evaluated this complex cell death mechanism by studying well-known hallmarks of apoptosis such as the caspase-3 activity, the detection of translocated phosphatidylserine and the identification of cells with fragmentation or CC. From direct analysis of apoptotic cells, we previously reported higher apoptotic susceptibility in cultured fibroblasts from antipsychotic-naive first-onset schizophrenia patients compared with controls.^15^ In the present study we further show that the mentioned apoptotic mechanisms are related to brain markers, in particular with brain metabolites and brain volume.

We did not find differences in metabolite levels between FES and controls in the AC or in the LT, which is in contrast with some studies involving antipsychotic-naive FES and might be related to our limited sample size. Such studies have usually found increased Gln levels in both AC and thalamus in never-treated patients with FES or subjects at high risk for psychosis,^7, 22, 23, 24, 25^ although lower^7, 26^ and negative results have also been reported.^27, 28^ Nevertheless, we found a relationship between decreased glutamatergic and NAA levels in the AC and increased cells with CC after both apoptosis inductions with STS. This finding supports the notion of an excitotoxic process, consistent with the idea that excitotoxicity occurs primarily in the early stages of the illness.^19^ It has been proposed that levels of Glu and NAA would decrease over the course of the illness, consistent with reports of decreased levels of both Glu and Gln in the AC in patients with chronic schizophrenia^29, 41, 42^ Aoyama *et al.*^29^ reported NAA and Glu reductions between 10- and 80-month assessments in schizophrenia patients, and both metabolites tended to be positively correlated in the AC, which is consistent with our results. Decline of NAA levels, which are thought to reflect neuronal integrity, would be consistent with decreasing glutamatergic metabolite levels, suggesting that both metabolites may reflect neuropil loss or altered apoptosis. A recent meta-analysis by Marsman *et al.*^43^ provided additional support for a progressive decrease in frontal Glu and Gln in patients with schizophrenia, possibly indicating a progressive loss of synaptic activity. Although the results in the LT did not overcome Bonferroni correction, the inverse relationship between caspase-3 activity (both baseline and after apoptosis induction with STS) and glutamatergic levels is also consistent with the reported data in the AC. Caspase-3 is an important apoptosis-inducing molecule acting in final stages of this cell death program,^15^ which has been suggested to increase transiently around the onset of psychosis and later downregulate as an appropriate long-term compensatory response to earlier stress^13^ and may be due to second-generation antipsychotics, which seem to be more neuroprotective than the first generation by upregulating antiapoptotic proteins.^44, 45^

With regard to brain volume, there was no significant difference between FES and controls but the number of cells showing CC after both apoptosis inductions with STS were negatively correlated to the right superior temporal cortex and postcentral areas in both groups. This suggests that cells that may experience an increased apoptotic susceptibility may correlate with volumetric measures in these brain regions. This finding is also consistent with studies showing loss of Glu and Gln related to GM loss in subjects with schizophrenia or at high risk of developing the disease.^24, 29^ This approach may help to reflect specific populations of neurons that may be more vulnerable to apoptosis and contribute to the onset of schizophrenia.^46^ Several post-mortem studies have examined spine density changes in brain regions showing the greatest indices of GM loss in schizophrenia, and these results support the view that spine density changes directly contribute to GM loss in the disease.^20, 47^ A reduction in the superior temporal gyrus GM volume is one of the most consistently reported alterations in the schizophrenia brain.^48^ At the cellular level, patients with schizophrenia show a profound reduction in spine density on pyramidal neurons in the superior temporal gyrus.^49^ Gray matter volume covariance changes have also been described in insula, amygdala and postcentral gyrus in first-episode treatment-naive schizophrenia patients.^50^ Although post-mortem studies cannot identify the root cause of spine loss, it is likely that spine formation and stability are reduced or spine pruning is accelerated in schizophrenia.^51, 52^

Although results involved patients and controls, it is important to bear in mind that a greater percentage of cells undergoing apoptosis was detected in samples from patients with FES compared with controls.^15^ Therefore, in this study we have revealed that peripheral apoptosis markers, which are increased in FES patients, may correlate with brain markers, such as metabolites and regional brain volume, providing further data supporting neuroprogression in the early stages of schizophrenia. Considering that the correlations were only present in the schizophrenia group when the analysis was performed by group, we postulate that such correlations are probably more pronounced in these subjects compared with healthy controls. However, the current sample size does not allow us to test further this hypothesis.

Some potential limitations of the present study must be discussed at this point. We acknowledge that the relatively small number of subjects may have affected the sensitivity of our measures and prevented the detection of differences in Glx and NAA levels and regional brain volume between patients and controls. Furthermore, it may have limited the detection of further correlations between other apoptotic biomarkers and neuroimaging brain markers. However, the strength of our observed findings after appropriate corrections instills confidence in their validity. As we used a 3-Tesla scanner, we chose not to study Gln and Glu metabolites separately, which hampers comparison among studies. Studies reporting both metabolites have generally employed 4-Tesla scanners.^23, 41^ One significant limitation of the current approach is that the apoptotic profile of peripheral fibroblasts does not necessarily reflect that of cortical brain tissue. As we are relating a peripheral experimental model for investigating apoptosis expected in neurons with central neuroimaging brain markers, results should be interpreted as exploratory and need to be replicated. However, this complex and useful model for studying neurodevelopmental and neurodegenerative diseases^53, 54, 55^ provides valuable and novel data that highly contribute to the understanding of the mechanisms that may underlie neuroprogression in schizophrenia. In addition, the longitudinal 1H-MRS data and biopsies that we are collecting in this sample will help to clarify whether the observed relationships between apoptotic peripheral and brain neuroimaging markers change over time.

Overall, the present results expand our previous data of increased apoptosis susceptibility in first-episode antipsychotic-naive schizophrenia patients compared with controls revealing for the first time that peripheral markers of apoptosis obtained in cultured fibroblasts may correlate with brain metabolites, Glx and NAA, and regional brain volume, which is consistent with neuroprogression in the early stages of schizophrenia.

## Figures and Tables

**Table 1:** Demographic and clinical data of subjects.

	*Fibroblast cell lines and structural MRI data*		P*-value*	*1H-MRS*		P*-value*
	*Patients (*N=*11)*	*Controls (*N=*7)*		*Patients (*N=*10)*	*Controls (*N=*6)*	
Age (mean±s.e.m.)	23.5±1.1	22.8±0.9	0.675	23.9±1.2	22.3±0.8	0.373
Male gender, *N* (%)	6 (54.5)	4 (57.1)	0.914	5 (50.0)	3 (50.0)	1.000
Caucasian ethnicity, *N* (%)	10 (90.9)	7 (100)	0.412	9 (90.0)	6 (100)	0.424
BMI (mean±s.e.m.)	21.3±1.2	21.4±0.4	0.913	20.3±0.7	21.4±0.4	0.248

*Educational level*^a^						
Post-compulsory schooling^b^, *N* (%)	9 (81.8)	7 (100)	0.231	8 (80.0)	6 (100)	0.242
University studies, *N* (%)	1 (9.1)	1 (14.3)	0.732	1 (10.0)	0 (0.0)	0.424

*Tobacco use*						
* N* (%)	6 (54.5)	2 (28.6)	0.280	5 (50.0)	2 (33.3)	0.515
No. of cigarettes per month	250.9±81.1	128.6±89.2	0.327	231.0±87.0	150.0±102.5	0.566
Sporadic cannabis use, *N* (%)	5 (45.5)	3 (42.9)	0.914	4 (40.0)	2 (33.3)	0.790
Sporadic alcohol use, *N* (%)	9 (81.8)	5 (71.4)	0.605	8 (80.0)	4 (66.7)	0.551

*Psychopathology score*						
PANSS total (mean±s.e.m.)	117.0±6.2	—	—	117.4±6.9	—	—
PANSS positive (mean±s.e.m.)	28.7±1.3	—	—	28.6±1.4	—	—
PANSS negative (mean±s.e.m.)	29.6±2.3	—	—	30.2±2.5	—	—
PANSS general (mean±s.e.m.)	58.7±4.1	—	—	58.7±4.5	—	—
CGI total (mean±s.e.m.)	5.3±0.3	—	—	5.3±0.3	—	—
GAF (mean±s.e.m.)	22±2.8	—	—	21±3.0	—	—

Abbreviations: BMI, body mass index; CGI, clinical global impression; GAF, global assessment of functioning; 1H-MRS, proton magnetic resonance spectroscopy; MRI, magnetic resonance imaging; PANSS, Positive and Negative Syndrome Scale.

a Subjects who completed the corresponding level.

b Spanish baccalaureate or vocational studies.
